# An unusual association of diffuse adenomyomatosis with dysplastic adenoma in chronic calculous cholecystitis: case presentation

**DOI:** 10.1186/1471-230X-10-41

**Published:** 2010-04-27

**Authors:** Isidoro Di Carlo, Adriana Toro, Elia Pulvirenti, Monica Zisa, Antonio Galia

**Affiliations:** 1 Department of Surgical Sciences, Organ Transplantation and Advanced Technologies, University of Catania, Cannizzaro Hospital, Catania, Italy; 2 Department of Pathology, Cannizzaro Hospital, Catania, Italy

## Abstract

**Background:**

Gallbladder adenomyomatosis is an epithelial proliferation and hypertrophy of the muscularis mucosae of the gallbladder. Rokitansky-Aschoff sinuses are a characteristic of this condition. The segmental adenomyomatosis has a higher risk of developing into gallbladder carcinoma, especially in the fundal region of elderly patients.

We report the case of a patient affected by chronic calculous cholecystitis with diffuse adenomyomatosis associated with dysplastic adenoma.

**Case presentation:**

An 81-year-old woman presented at our hospital with a 1-year history of intermittent pain localized at the right upper abdominal quadrant, without diffusion to any other body part. On physical examination the abdomen was soft, not distended, and tender to palpation in the right upper quadrant. Murphy sign was negative. Laboratory tests were normal. The patient was scheduled for a laparoscopic cholecystectomy, and neither endoscopic ultrasonographic scan nor magnetic resonance imaging was performed. The operation, performed after obtaining informed consent, was uncomplicated and the intra-operative pathological examination showed no malignancy. The definitive pathological examination of the gallbladder showed: multiple stones of cholesterol origin; diffuse mucosal adenomyomatosis; and a 1.1 cm pedunculated mass localized at the fundus, whose surface was lumpy. This mass was diagnosed as an adenoma with multiple areas of severe dysplasia.

**Conclusions:**

The adenoma of the gallbladder, together with the dysplasia, represents a biological carcinogenetic model. Carcinoma has rarely been reported in adenomyomatosis. Degenerative risk suggests surgery should be mandatory when there is a concomitant presence of large adenoma and adenomyomatosis.

## Background

Gallbladder adenomyomatosis (GBA) is defined as an epithelial proliferation and hypertrophy of the muscularis mucosae of the gallbladder with outpouching of the mucosa into or through the thickened muscular layer, forming the so called Rokitansky-Aschoff sinuses, which are a characteristic of this condition [1]. Gallbladder adenomas are benign tumors usually found as polyps; they are relatively uncommon, ranging from 0.3% to 0.5% of gallbladders removed by cholecystectomy [2].

Segmental adenomyomatosis has a higher risk of developing into gallbladder carcinoma, especially in the fundal region of elderly patients [3]. Chronic calculous cholecystitis with dysplastic adenoma associated with diffuse adenomyomatosis is not found in literature, and its discovery may be due to laparoscopic cholecystectomy greatly increased in the last two decades. We report the case of a patient affected by chronic calculous cholecystitis with dysplastic adenoma associated with diffuse adenomyomatosis.

## Case presentation

An 81-year-old woman presented at our hospital with a 1-year history of intermittent pain localized at the right upper abdominal quadrant, without diffusion to any other body part. The patient denied episodes of fever during the previous months. Her medical history was significant for hypertension, transient ischemic attack (TIA), and sigmoid diverticulitis.

The patient was alert and oriented during physical examination. The abdomen was soft, not distended, and tender to palpation in the right upper quadrant. Murphy sign was negative.

Laboratory tests were normal. Ultrasonographic scan (US) of the right upper abdominal quadrant showed multiple gallstones. A heterogeneous, hypoechogenic pedunculated mass, 1.1 cm in diameter, was found in the fundus of the gallbladder. The patient underwent an upper gastrointestinal endoscopy (UGIE) that showed normal gastro-duodenal mucosa. Colonoscopy showed a small polyp, which was removed. The patient was scheduled for a laparoscopic cholecystectomy. Endoscopic US and magnetic resonance imaging (MRI) were not performed. The operation was uncomplicated and the intra-operative pathological examination showed no malignancy. The postoperative course was unremarkable and the patient was discharged on the first postoperative day.

**Figure 1:**
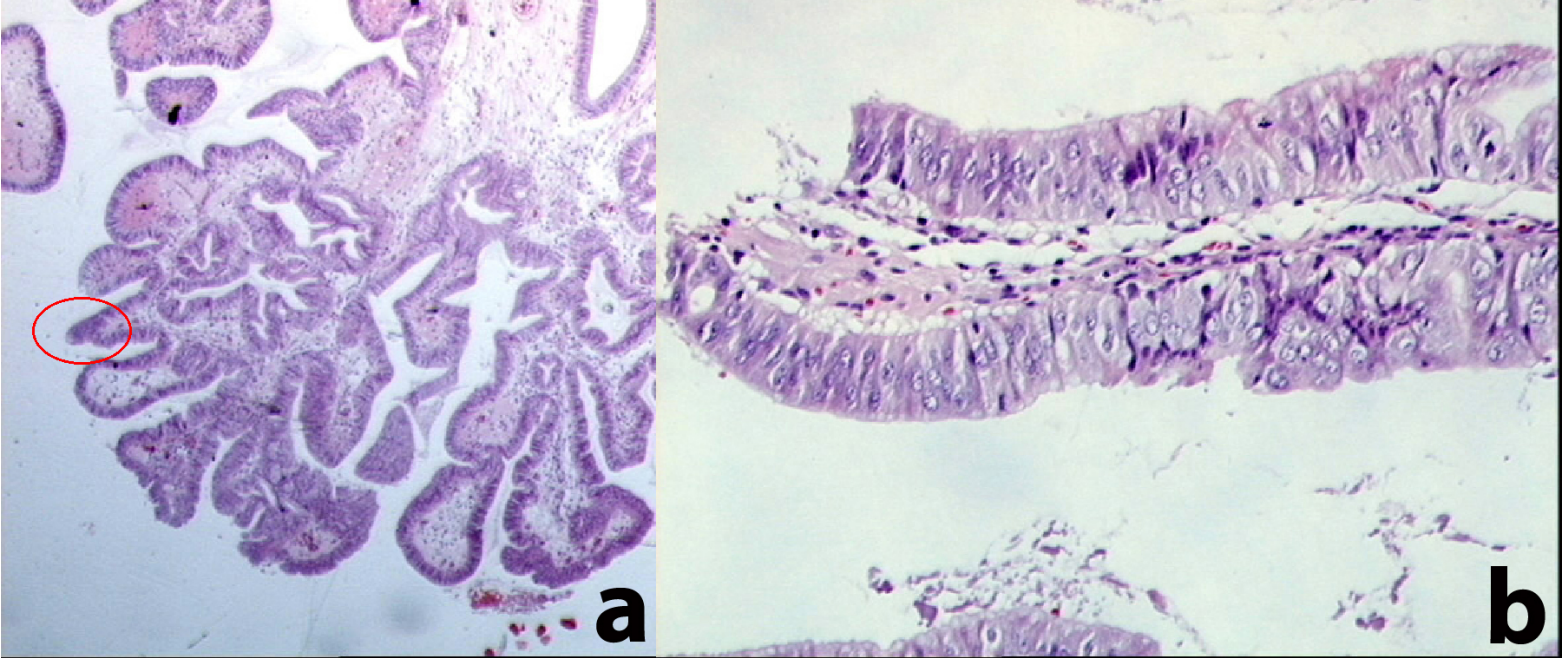
Diffuse mucosal adenomyomatosis with zones of dysplasia (red circle in a), with the same image at high enlargement (b)

**Figure 2:**
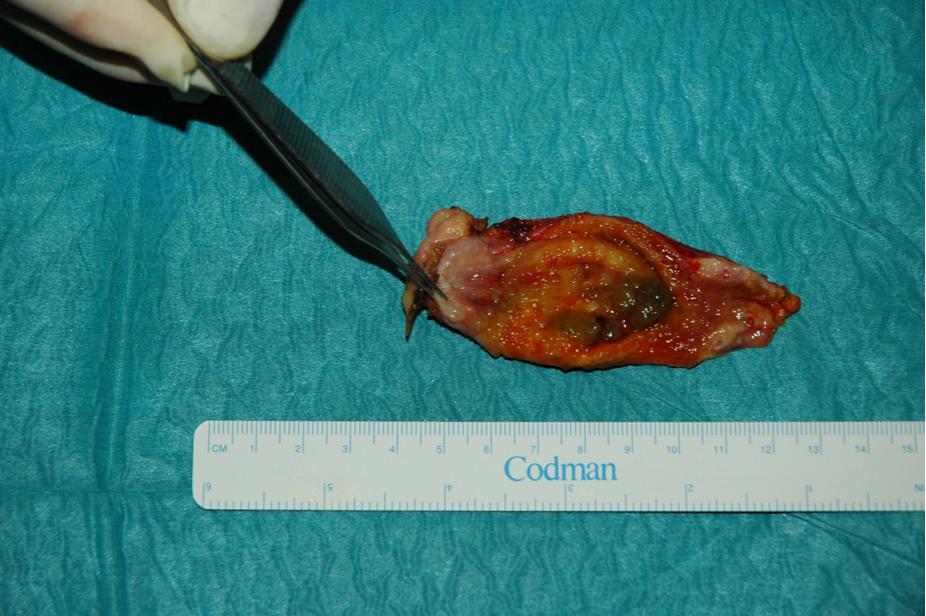
**Mass localized at the fundus of the gallbladder (adenoma)**.

**Figure 3:**
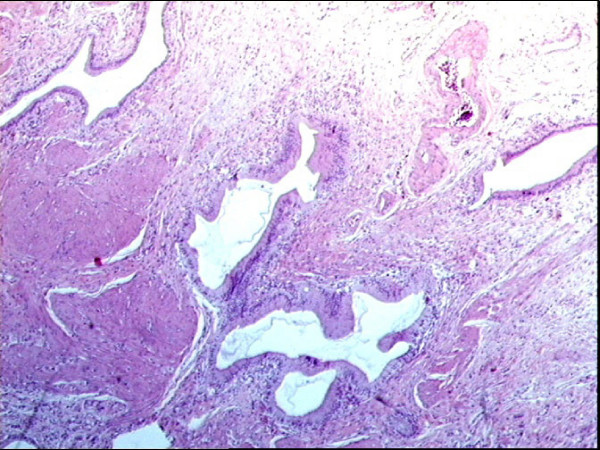
**Adenomyomatosis (prominent R-A sinus) of diffuse type**.

**Figure 4:**
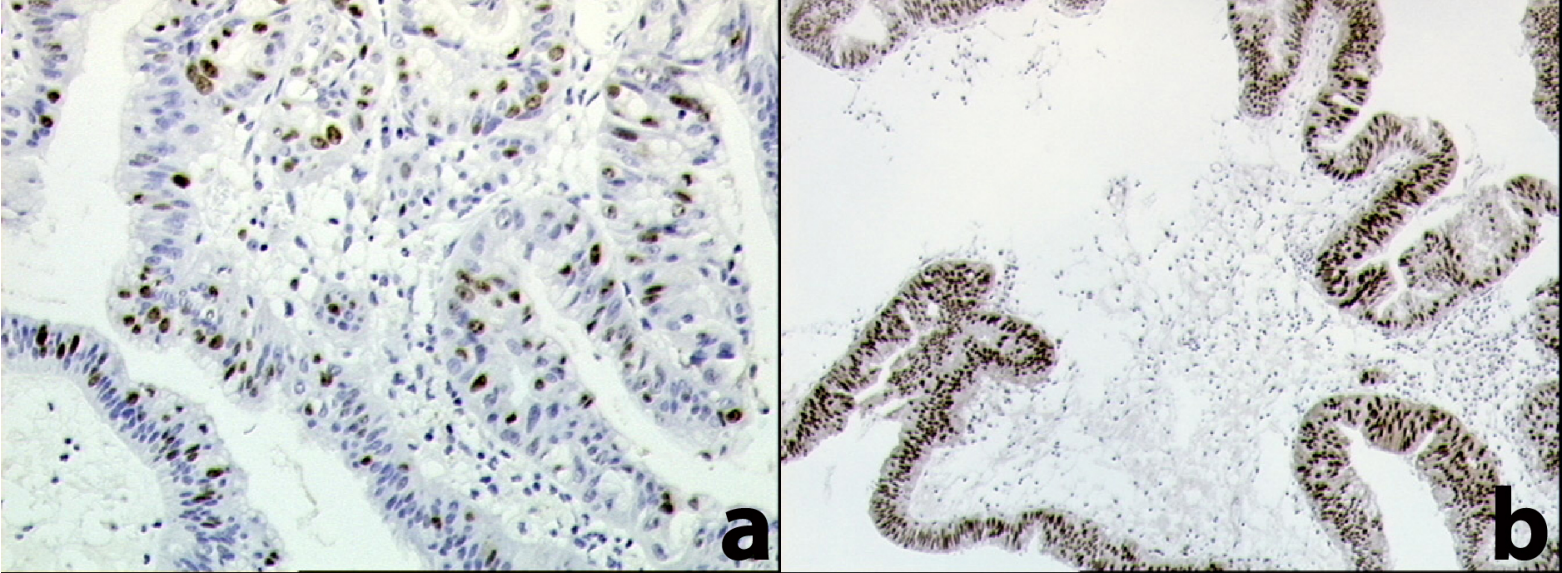
**Immunohistochemical analysis**. a: high proliferative index estimate with ki-67 (Mib-1 antibody); b: strong and diffuse immunoreactivity for p53 protein.

The definitive pathological examination of the gallbladder showed: multiple stones of cholesterol origin; diffuse mucosal adenomyomatosis (Figure 1); and a 1.1 cm pedunculated mass localized at the fundus (Figure 2), whose surface was lumpy. The mass was diagnosed as an intestinal type adenoma with mixed tubular and villous features and multiple areas of severe grade dysplasia, present as nuclear enlargement, high nuclear to cytoplasmatic ratio, nuclear hyperchromasia, and large nucleoli (Figure 3). Immunohistochemical analysis showed a pre-malignant lesion of the gallbladder (Figure 4).

## Conclusion

Polyps of the gallbladder can be differentiated into non-neoplastic and neoplastic lesions [4]. They can be solitary or multiple; pedunculated or sessile; and they may have smooth, granular, or nodular surfaces. Non neoplastic polyps can show a cholesterol origin, as a result of excess lipid deposits in the gallbladder epithelium, or an inflammatory origin, as a consequence of chronic cholecystitis; the latter are usually composed of granulation and fibrous tissue [5].

Tumoral polyps may be classified as adenomas and adenocarcinomas. Sixty percent of gallbladder adenomas are associated with cholecystolithiasis [6]. There is little data related to whether adenomas represent pre-malignant lesions. One study, which followed 109 asymptomatic patients affected by polypoid lesions by US once or twice a year, identified only one case of gallbladder carcinoma [7]. On the contrary, other studies demonstrated large polyps (>1 cm) are frequently malignant [4,6]. The incidence of malignant tumors increases significantly in lesions 10 mm or greater in size, and a considerable number of lesions 15 mm or greater are malignant [8].

Adenomyomatosis is an acquired, benign proliferative lesion of the gallbladder characterized by mucosal proliferation with invaginations and diverticula penetrating into the thickened muscular layer (Rokitansky-Aschoff sinuses) [9]. Adenomyomatosis consists of three types: focal, segmental, and diffuse [10]. Segmental adenomyomatosis has a higher risk of developing into gallbladder carcinoma, especially in the fundal region of elderly patients [10]. Nabatame et al. reported a higher incidence of epithelial metaplasia in the fundal mucosa of segmental adenomyomatosis than in the neck mucosa [10], with an associated risk of increased carcinogenesis.

In our patient, we found an adenoma with signs of severe dysplasia inside areas of diffuse adenomyomatosis. This has not been reported and could represent a different risk for carcinogenesis. Chronic calculous cholecystitis with dysplastic adenoma associated with diffuse adenomyomatosis is not found in literature, and its discovery may be due to laparoscopic cholecystectomy, which has been increasingly used in the last two decades.

Patients with adenomyomatosis and (or without) adenomas may present signs or symptoms of chronic cholecystitis or acute-on-chronic cholecystitis such as right upper quadrant pain, nausea, vomiting associated with meals, or more unexplained signs [11]. However, the majority of patients are asymptomatic and the diagnosis of adenoma is usually rare. Our patient was symptomatic and clinical signs were probably related to lithiasis, but their relationship to adenoma or adenomyomatosis cannot be excluded.

US is useful to diagnose adenoma and adenomyomatosis, and could be sufficient in patients that should be treated surgically for symptoms or for the dimensions of adenomas (>1 cm in diameter). In asymptomatic patients with lesion smaller than 1 cm, MRI or ecoendoscopic ultrasonography (EUS) may be used to obtain a differential diagnosis [12]. EUS may be useful to differentiate non neoplastic from neoplastic polyps of the gallbladder; however, caution should be used because some findings can also occur in neoplastic polyps when they contain a concomitant non neoplastic component (cholesterosis or proliferated Rokitansky-Aschoff sinuses). MRI may provide important information for the diagnosis of adenomyomatosis and may differentiate it from gallbladder carcinoma. On MRI, these sinuses appear as small intramural foci of low T1 and high T2 signal intensity. Early linear mucosal enhancement is seen, which turns homogeneous on late phases. On MRI images, the tumor usually shows T1 and high T2 signal intensity. The enhancement pattern of gallbladder carcinoma is described as irregularly delineate peripheral enhancement in early phases and further enhancement in late phase [13]. Due to the small number of patients studied up to now, this procedure is not well validated and should be confirmed by other studies [14].

Our patient only underwent US due to her symptoms, and was promptly operated on. Surgery should be performed quickly when symptoms are present. In asymptomatic patients, laparoscopic surgery may become mandatory if diagnosis remains uncertain after EUS or MRI. Moreover, as in this case report, degenerative risk may exist in non-reported conditions, suggesting surgery should be mandatory when there is a concomitant presence of large adenoma and adenomyomatosis.

## Abbreviations

**GBA**: gallbladder adenomyomatosis; **TIA**: transient ischemic attack; **US**: ultrasonographic scan; **UGIE**: upper gastrointestinal endoscopy; **MRI**: magnetic resonance imaging; **EUS**: ecoendoscopic ultrasonography.

## Consent

Written informed consent was obtained from the patient for publication of this case report and any accompanying images. A copy of the written consent is available for review by the Editor-in-Chief of this journal.

## Competing interests

The authors declare that they have no competing interests.

## Authors' contributions

IDC: designed the research, carried out the study, drafted the manuscript, and also performed the operations. AT: contributed to this work by writing the paper and performing the literature research. EP: contributed to this work by writing the paper and performing the literature research. MZ: performing the literature research. AG: performed the pathologic study. All Authors read and approved the final manuscript.

## Pre-publication history

The pre-publication history for this paper can be accessed here:

http://www.biomedcentral.com/1471-230X/10/41/prepub
